# Prostate Cancer Susceptibility Loci Identified in *GATA2* and *ZMIZ1* in Chinese Population

**DOI:** 10.1155/2022/8553530

**Published:** 2022-03-24

**Authors:** Hui-Jing Zhang, Zhongyuan Liu, Liang Kan

**Affiliations:** ^1^Department of Endoscopy, The First Affiliated Hospital of China Medical University, Shenyang, Liaoning, China; ^2^Department of Geriatrics, Shengjing Hospital of China Medical University, Shenyang, China

## Abstract

**Background:**

Common genetic risk variants for prostate cancer (PCa) have been identified at approximately 170 loci using genome-wide association studies (GWAS), most of which were identified in European populations. Recently, GWAS were applied to a large Japanese cohort and identified 12 novel susceptibility loci associated with PCa risk. In this study, we aim to investigate PCa susceptibility loci in the Chinese population. The study data will be used to promote PCa risk control in China.

**Methods:**

A total of 235 PCa patients and 252 control subjects (all unrelated) were enrolled in this case-control PCa study. Nine single nucleotide polymorphisms (SNPs) were genotyped in *GATA2* (rs73862213, rs2335052, and rs10934857), *ZMIZ1* (rs704017, rs77911174, and rs3740259), and *SUN2* (rs78397383, rs5750680, and rs138705) genes. The associations between the candidate SNPs and PCa were analyzed using multiple-factor logistic regression and haplotype analysis.

**Results:**

The allele frequency distributions of rs73862213 and rs2335052 in the *GATA2* gene and rs704017 and rs77911174 in the *ZMIZ1* gene were found to be significantly different between PCa cases and controls. Haplotype analysis revealed that the G-C-A haplotype of the *GATA2* gene (order of SNPs: rs73862213-rs2335052-rs10934857) and the G-G-G haplotype of the *ZMIZ1* gene (order of SNPs: rs704017-rs77911174-rs3740259) were associated with increased PCa risk. None of the *SUN2* haplotypes were associated with PCa.

**Conclusions:**

Our study data indicates that the minor alleles of rs73862213 and rs2335052 in the *GATA2* gene and rs704017 and rs77911174 in the *ZMIZ1* gene were associated with increased PCa risk. These findings greatly extended our knowledge of the etiology of PCa.

## 1. Introduction

Prostate cancer (PCa) is one of the most frequently diagnosed malignant tumors among males worldwide. Accordingly, PCa is the leading cause of cancer-related deaths in men according to Global Cancer Statistics 2018 [1]. Although PCa incidence and mortality rates vary substantially by race, ethnicity, and geography, PCa incidence remains highest in countries defined as “developed” (e.g., North America, Western and Northern Europe, and Australia). Men from East, Southeast, and South Central Asia have the lowest rates of prostate cancer death [2]. However, recent studies showed that PCa incidence and morbidity have been rapidly increasing in China, especially in urban areas [3].

The large differences in the incidence of PCa among different ethnic groups suggest involvement of specific genetic risk factors in these cancer disparities. Numerous marker genes, including *HPC1*, *PCAP*, *HPCX*, *CAPB*, *HPC20*, and *HOXB13*, were identified in hereditary PCa. These genes were shown to contain about 170 PCa-related risk loci identified in different cohorts using genome-wide association study (GWAS) methods [4–6]. However, most of these studies were conducted using data collected in populations of European ancestry or cohorts with mixed ethnicity. Large-scale GWAS was recently performed using an independent Japanese cohort [7]. The study confirmed the association between several previously reported loci and PCa risk in the Japanese population. Additionally, 12 novel PCa-linked susceptibility loci were identified, including rs1125927 (*TMEM17*), rs73862213 (*GATA2*), rs77911174 (*ZMIZ1*), and rs138708 (*SUN2*). Seven of the reported loci had particularly low minor allele frequency in European population [7].

Although the *GATA2*, *ZMIZ1*, and *SUN2* gene-located loci were reported as risk markers for PCa in the Japanese cohort, the association between these genes and PCa was not tested in a Chinese population. In this replication study, the expression of nine single nucleotide polymorphisms (SNPs) of *GATA2*, *ZMIZ1*, and *SUN2* genes, including previously reported rs73862213 (*GATA2*) and rs77911174 (*ZMIZ1*), was investigated as a potential PCa risk factor in the Chinese population.

## 2. Material and Methods

### 2.1. Study Subjects

In this study, a total of 235 PCa patients and 252 normal controls were recruited from the Shengjing Hospital of China Medical University from June 2014 to July 2019. The study protocol was approved by the human ethics committee of the Shengjing Hospital, China Medical University. All study participants signed the informed consent. The PCa diagnosis was confirmed by two experienced pathologists according to the prostate cancer guidelines of the Chinese Society of Clinical Oncology (CSCO). A comprehensive examination was performed in all participants. All recruited patients completed the approved study questionnaire. The control subjects with a family history of cancer were excluded. The clinical characteristics, including patient's age, level of prostate specific antigen (PSA), body mass index (BMI), Gleason score, and prognostic stage (according to the AJCC 8th Edition Cancer Staging Form), were recorded for all enrolled participants. Peripheral blood sample (5 mL) anticoagulated with EDTA was collected from all patients (both control and PCa groups).

### 2.2. Selection of SNPs and Genotype

Three PCa risk-related candidate genes (*GATA2*, *ZMIZ1*, and *SUN2*) were analyzed in this study. The genes were genotyped in a Chinese Han population in Beijing (CHB). The genotype data was downloaded and analyzed using Ensembl Project public data site (http://asia.ensembl.org/Homo_sapiens/Tools/VcftoPed?db=core). Loci with more than two alleles were excluded. SNP selection was conducted according to the following criteria: (a) SNPs with a potential role in regulation of translational functions (variants located in the 5′ or 3′ untranslated regions (UTRs) that may affect the splice of mRNA or missense variants that lead to a protein modification); (b) SNPs with minor allele frequency (MAF) > 0.05; and (c) SNPs that were reported as PCa risk-associated in the Japanese GWAS study 7. Thus, altogether, 9 SNPs from *GATA2*, *ZMIZ1*, and *SUN2* candidate genes were included and analyzed in this study (Table 1).

Genomic DNA was isolated from all patients using a TIANamp Genomic DNA Kit (Tiangen Biotech, Beijing, China). Validation genotyping was performed using the TaqMan SNP genotyping assay and 7500 Real-Time PCR System (Applied Biosystems, Foster City, CA, USA) according to the manufacturer's instructions. The PCR reactions were conducted in a final volume of 10 *μ*L amplification mix, including 5 *μ*L of master mix (Applied Biosystems), 0.5 *μ*L predesigned PCR primers and probes (Applied Biosystems), 50 ng gDNA, and ddH_2_O. Amplification was performed under the following conditions: an initial denaturation at 95°C for 10 min, followed by 40 cycles of denaturation at 95°C for 15 min and annealing at 60°C for 1 min.

### 2.3. Statistical Analysis

Statistical analyses were performed using SPSS software (IBM-SPSS, version 22.0, Chicago, IL, USA). The demographic characteristics of the study subjects, including age, PSA, and BMI, were compared using independent samples *t*-test between case and control groups. The Hardy-Weinberg equilibrium test for each SNP in the control group was calculated. Genotypic association between each SNPs and PCa was analyzed using multiple-factor logistic regression test. Odds ratios (OR) and 95% confidence intervals (95% CIs) were calculated using the major genotype as the reference genotype after data adjustments for age and BMI. SHEsis statistical analysis (http://analysis.bio-x.cn/myanalysis.php) was used to estimate the association between haplotypes and PCa.

## 3. Result

### 3.1. Analysis of Demographic Characteristics of Study Participants

The demographic characteristics of 235 PCa patients and 252 controls are presented in Table 2. The mean ages of case and control groups were 69.43 ± 9.33 and 68.37 ± 8.47, respectively. No statistically significant differences in age between the two groups were found (*p* = 0.238). Furthermore, no statistically significant differences in reported BMI were detected between case and control groups (*p* = 0.352). The serum PSA levels, a diagnostic factor of PCa, were significantly higher in PCa patients than controls (35.53 ± 53.74 vs. 8.76 ± 9.25, *p* < 0.001).

### 3.2. Genotype Distribution of SNPs

The Hardy-Weinberg equilibrium was estimated for each SNP in the control group. No deviations were observed (Table 1). Genotype distributions of candidate SNPs are shown in Table 3.

Significant association between *GATA2* gene SNPs (rs2335052 and rs73862213) and PCa was detected. The individuals with CT (*p* = 0.038), TT (*p* = 0.029), and CT + TT (*p* = 0.015) genotypes of rs2335052 had a higher risk of PCa compared with the CC genotype. There was no GG genotype of rs73862213 detected in both case and control groups. However, the frequency of the AG genotype is significantly higher in cases than controls, indicating that the rs73862213 was associated with PCa risk.

For the *ZMIZ1* gene, homozygotes of both rs77911174 and rs704017 were significantly associated with the risk of PCa, whereas no significant association was detected between the heterozygote genotype of these SNPs and the occurrence of PCa (*p* = 0.129 and 0.066, respectively). The dominant allele model for both rs77911174 and rs704017 demonstrated significant associations with PCa (AG + GG vs. AA, *p* = 0.032 and 0.020, respectively). No association with PCa was observed for rs10934857 of *GATA2*, rs3740259 of *ZMIZ1*, and the three SNPs of *SUN2*.

### 3.3. Haplotype Analysis of *GATA2*, *ZMIZ1*, and *SUN2* Gene

The haplotypes of *GATA2*, *ZMIZ1*, and *SUN2* genes were analyzed, and data is shown in Table 4. The G-C-A haplotype (order of SNPs: rs73862213-rs2335052-rs10934857) of the *GATA2* gene, which was constructed with the minor allele of rs73862213, was significantly associated with the higher risk of PCa (*p* = 0.001). The individuals with the G-G-G haplotype of the *ZMIZ1* gene (order of SNPs: rs704017-rs77911174-rs3740259) demonstrated an increased PCa risk; whereas the A-A-G haplotype was associated with a decreased PCa risk (both associations were considered to be significantly associated with PCa; *p* < 0.01). There was no association between all *SUN2* haplotypes and PCa (*p* > 0.05).

## 4. Discussion

PCa has a considerable underlying genetic basis. It has been reported using candidate-gene analysis (or GWAS) that a number of genetic loci are associated with PCa. A recent GWAS-based analysis of the Japanese cohort reported 12 novel PCa-linked susceptibility loci, which are different from the PCa-related loci identified in European populations. In this study, we discussed the associations between the candidate SNPs in *GATA2*, *ZMIZ1*, and *SUN2* genes and PCa in the Chinese population.

*GATA2* is one of the six members of the GATA transcription factor family that regulates cell differentiation. The gene is located on chromosome 3q21.3. The *GATA2* gene has been associated with a variety of diseases, including coronary heart disease [8, 9], Parkinson's disease [10], and lung [11], colon [12], and prostate cancers [13]. As a transcription factor, *GATA2* plays an important role in the regulation of androgen receptor (AR) signaling [14]. Previously, Liu et al. [12] concluded that the carriers of the SNP rs2335052 minor allele are exposed to a significantly increased risk of recurrent colorectal cancer and indicated reduced disease-free survival rate. In this study, 3 loci (rs73862213, rs2335052, and rs10934857) of the *GATA2* gene were analyzed to test the association between these SNPs and PCa in the Chinese population. The results showed that heterozygous genotypes of rs73862213 were higher in the case than in the control group, indicating that the AG genotype is associated with PCa risk. Besides, the rs2335052 minor alleles correlated with the higher risk of PCa. Additionally, the haplotype analysis revealed a higher frequency of the G-C-A haplotype, which contained the G allele in rs73862213 of *GATA2*, in the case group compared to controls. This finding confirms that *GATA2* may be a potential PCa-causing candidate gene. Neither the G-T-G nor the G-T-A, constructed with the minor allele of rs73862213 and rs2335052, were detected in this study because of the lower allele frequency and limited sample number.

The *ZMIZ1* (zinc finger MIZ-type containing 1) gene is located on 10q22.3 chromosome and encodes the PIAS-like protein containing 1067 amino acid residues. *ZMIZ1* belongs to the protein inhibitor of activated STAT (PIAS) family. The protein contains a ring finger region termed Miz, a nuclear localization sequence (NLS), and two proline-rich regions [13]. *ZMIZ1* is an established transcriptional coactivator which can increase transcriptional activity of other DNA-binding factors, including AR, SMAD3, STAT, and p53. It has been shown that *ZMIZ1* colocalizes with AR and enhances sumoylation of AR *in vivo*. Modified *ZMIZ1* signaling was associated with various diseases, including vitiligo [13], multiple sclerosis [15], leukemia [16], prostate cancer [17], and inflammatory bowel disease [18]. Multiple studies reported that rs704017, located in the intron of the *ZMIZ1* gene, is the genetic risk factor associated with colorectal cancers (CRC).

The rs704017 SNP is located in the intron 3 of *ZMIZ1* antisense RNA 1 (*ZMIZ1*-AS1, a miscellaneous RNA (miscRNA)), residing in a strong enhancer region. A recent large-scale genetic study in an East Asian population defined this SNP as a risk factor for CRC [17]. Several GWAS conducted in Spanish [19], Japanese [20], and Han Chinese populations [21] supported this finding. Tan et al. [21] found that rs704017 is significantly associated with stage III or IV CRCs. Song et al. reported [22] that rs704017 was also associated with disease-free survival (DFS) in CRC patients. Consistently, the risk allele rs704017 was less frequently detected among the younger CRC patients (age < 50 years) compared to the older CRC patients (age ≥ 50 years), suggesting that rs704017 has a hereditary effect on CRC onset age [23].

In this study, analysis of three SNPs located in the *ZMIZ1* gene showed that *ZMIZ1* is a candidate PCa risk factor gene. The patients with GG homozygotes of rs77911174 and rs704017 had a higher risk of PCa than those with AA homozygotes. The same risk associations were observed for the dominant allele model (both AG + GG vs. AA, *p* < 0.05). However, for the heterozygote genotypes of the two SNPs, no significant differences in the occurrence of PCa between the case and control groups were detected (*p* = 0.129 and 0.066, respectively). Interestingly, the haplotype analysis demonstrated that the individuals with the G-G-G haplotype of *ZMIZ1* (order of SNPs: rs704017-rs77911174-rs3740259), constructed with both minor allele of rs77911174 and rs704017, had an increased PCa risk (*p* < 0.01). Alternatively, individuals with the A-A-G haplotype had a decreased risk of PCa (*p* < 0.01). The results of genotype distributions and haplotype analysis suggested that the *ZMIZ1* gene may be related with the occurrence of PCa.

*SUN2* (SAD1/UNC84 domain protein-2) is the key component of the linker of nucleoskeleton and cytoskeleton (LINC) complex which plays an important role in nuclear-cytoplasmic connections [24]. The role of *SUN2* as cancer suppressor was demonstrated in various cancers, including embryonal tumors [25], breast cancer [26], lung cancer [27], and PCa [28]. Moreover, *SUN2* was found to enhance the chemotherapy sensitivity in lung cancer cells exposed to cisplatin. Higher *SUN2* level predicts a better overall survival in lung cancers [27]. Recently, rs138708 in *SUN2* (nonsynonymous SNP) was reported as PCa risk susceptibility loci in a Japanese population [7]. However, in this study, we did not observe the association between the three SNPs in *SUN2* and PCa in the Chinese population. These findings are inconsistent with those data reported in Takata et al.'s study. Different populations and limited samples may contribute these discrepancies.

There are several limitations in our study. Firstly, the sample size is relatively small. A multicenter study with a large sample size may be more convincing. The small sample size is potentially the reason that we failed to replicate the association between the *SUN2* gene and PCa. Secondly, we did not investigate the other susceptibility genes reported in the Japanese GWAS. Thirdly, we did not perform function-related studies using candidate genes in this study. The functional or biological significance related to PCa development is still unclear. Additionally, gene-to-gene and gene-to-environment interactions were not analyzed.

In summary, nine loci in *GATA2*, *ZMIZ1*, and *SUN2* genes were studied in the Chinese population. Our data reported four important PCa susceptibility loci that have not been previously concerned in China. The results of our study indicate that rs73862213 and rs2335052 in the *GATA2* gene and rs77911174 and rs704017 in the *ZMIZ1* gene may be independent indicators for PCa risk in the Chinese population. Further large-scale studies are required to validate these findings and clarify the tested SNPs underlying mechanism of signaling.

## Figures and Tables

**Table 1:** SNPs in the *GATA2*, *ZMIZ1*, and *SUN2* gene.

Gene	Band location	SNPs	Functional location	Allele	MAF	HWE *p* in the control group
*GATA2*	3q21.3	rs73862213	Upstream	A/g	0.078	0.208
		rs2335052	Exon4	C/t	0.398	0.134
		rs10934857	3′-UTR	G/a	0.136	0.089
*ZMIZ1*	10q22.3	rs704017	Upstream	A/g	0.291	0.756
		rs77911174	Upstream	A/g	0.335	0.103
		rs3740259	3′-UTR	G/a	0.248	0.496
*SUN2*	22q13.1	rs78397383	Upstream	A/c	0.252	0.448
		rs5750680	Upstream	C/t	0.432	0.685
		rs138705	Intron 17	C/t	0.481	0.107

**Table 2:** Demographic characteristics of study subjects.

	Cases (*n* = 235)	Controls (*n* = 252)	*p* values
Age	69.43 ± 9.33	68.37 ± 8.47	0.238
PSA	35.53 ± 53.74	8.76 ± 9.25	<0.001
BMI	23.76 ± 8.13	22.32 ± 8.56	0.352
Gleason score			
<6 (3 + 3)	86 (36.6%)	—	
7 (3 + 4, 4 + 3)	71 (30.2%)	—	
8 (4 + 4)	42 (17.9%)	—	
9-10 (4 + 5, 5 + 4, 5 + 5)	36 (16.3%)	—	
Prognosis stage			
Stage I	78 (33.2%)	—	
Stage II	85 (36.2%)	—	
Stage III	46 (19.6%)	—	
Stage IV	26 (11.0%)	—	

**Table 3:** Genotype distribution of SNPs in case and control groups.

Gene	rs No.	Genotype	Cases	Controls	OR (95% CI)	*p* value^∗^
*GATA2*	rs73862213	AA	181	215	Ref	Ref
		AG	54	37	1.734 (1.091-2.753)	0.018
		GG	0	0	—	—
		AG + GG	54	37	1.734 (1.091-2.753)	0.018
	rs2335052	CC	72	104	Ref	Ref
		CT	113	107	1.525 (1.022-2.276)	0.038
		TT	50	41	1.762 (1.057-2.935)	0.029
		CT + TT	163	148	1.591 (1.095-2.312)	0.015
	rs10934857	GG	166	186	Ref	Ref
		GA	57	57	1.120 (0.734-1.710)	0.598
		AA	12	9	1.494 (0.614-3.635)	0.374
		GA + AA	69	66	1.174 (0.788-1.742)	0.435
*ZMIZ1*	rs704017	AA	91	124	Ref	Ref
		AG	112	107	1.426 (0.977-2.083)	0.066
		GG	32	21	2.076 (1.124-3.834)	0.018
		AG + GG	144	128	1.533 (1.069-2.198)	0.020
	rs77911174	AA	92	123	Ref	Ref
		AG	99	98	1.351 (0.916-1.992)	0.129
		GG	44	31	1.898 (1.113-.3.234)	0.018
		AG + GG	143	129	1.482 (1.034-2.124)	0.032
	rs3740259	GG	123	146	Ref	Ref
		AG	90	89	1.200 (0.822-1.753)	0.344
		AA	22	17	1.536 (0.781-3.023)	0.212
		AG + AA	112	106	1.254 (0.877-1.794)	0.215
*SUN2*	rs78397383	AA	124	136	Ref	Ref
		AC	92	95	1.062 (0.729-1.547)	0.753
		CC	19	21	0.992 (0.510-1.933)	0.982
		AC + CC	111	116	1.049 (0.735-1.499)	0.790
	rs5750680	CC	67	79	Ref	Ref
		CT	119	127	1.105 (0.733-1.666)	0.634
		TT	49	46	1.256 (0.749-2.107)	0.388
		CT + TT	168	173	1.145 (0.776-1.689)	0.494
	rs138705	CC	72	75	Ref	Ref
		CT	115	113	1.060 (0.700-1.605)	0.783
		TT	48	64	0.781 (0.476-1.281)	0.328
		CT + TT	163	177	0.959 (0.651-1.413)	0.833

∗: adjusted for age and BMI.

**Table 4:** Association between haplotypes of three candidate genes and PCa.

Gene	Haplotype	Cases	Controls	OR (95% CI)	*p*
*GATA2*	A-C-G	107 (45.5%)	121 (48.0%)	0.853 (0.685-1.573)	0.247
	A-T-G	82 (34.9%)	94 (37.3%)	1.158 (0.835-1.867)	0.083
	G-C-A	31 (13.2%)	18 (7.14%)	1.642 (1.236-2.768)	0.001
	A-C-A	6 (2.55%)	10 (3.97%)	0.854 (0.638-1.769)	0.125
*ZMIZ1*	A-A-G	54 (22.9%)	71 (28.2%)	0.569 (0.427-0.874)	<0.01
	A-G-G	53 (22.5%)	62 (24.6%)	0.876 (0.644-1.527)	0.254
	G-A-G	44 (18.7%)	46 (18.2%)	1.023 (0.856-1.735)	0.538
	A-A-A	21 (8.94%)	27 (10.7%)	0.956 (0.628-1.837)	0.633
	G-A-A	19 (8.08%)	18 (7.14%)	1.201 (0.953-1.674)	0.573
	G-G-G	21 (8.94%)	8 (3.17%)	1.572 (1.248-2.639)	<0.01
*SUN2*	A-T-C	65 (27.7%)	74 (29.4%)	0.875 (0.673-1.864)	0.363
	C-C-T	42 (17.9%)	48 (19.1%)	0.924 (0.735-1.739)	0.246
	A-C-C	41 (17.4%)	42 (16.7%)	1.183 (0.679-1.536)	0.537
	A-C-T	33 (14.0%)	34 (13.5%)	1.045 (0.842-1.731)	0.438
	A-T-T	31 (13.2%)	32 (12.7%)	1.039 (0.742-1.825)	0.383

*GATA2*, order of SNPs: rs73862213-rs2335052-rs10934857. *ZMIZ1*, order of SNPs: rs704017-rs77911174-rs3740259. *SUN2*, order of SNPs: rs78397383-rs5750680-rs138705.

## Data Availability

The datasets used and/or analyzed during the current study are available from the corresponding author on reasonable request.
